# Knowledge, attitudes and practices towards yaws in endemic areas of Ghana, Cameroon and Côte d’Ivoire

**DOI:** 10.1371/journal.pntd.0012224

**Published:** 2024-06-20

**Authors:** Camila González Beiras, Adingra Tano Kouadio, Becca Louise Handley, Daniel Arhinful, Serges Tchatchouang, Ahouansou Stanislas Sonagnon Houndji, Eric Tettey Nartey, Dolphine Osei Sarpong, Gely Menguena, Philippe Ndzomo, Laud Anthony Basing, Kouadio Aboh Hugues, Ivy Brago Amanor, Mohammed Bakheit, Emelie Landmann, Patrick Awondo, Claudia Müller, Tania Crucitti, Nadine Borst, Lisa Becherer, Simone Lüert, Sieghard Frischmann, Aboubacar Sylla, Mireille S. Kouamé-Sina, Emma Michèle Harding-Esch, Sascha Knauf, Oriol Mitjà, Sara Eyangoh, Kennedy Kwasi Addo, Solange Ngazoa Kakou, Michael Marks

**Affiliations:** 1 Skin Neglected Tropical Diseases and Sexually Transmitted Infections section, Fight Infectious Diseases Foundation, Hospital Universitari Germans Trías i Pujol; Badalona, Spain; 2 Universitat Autònoma de Barcelona, Bellaterra, Spain; 3 Institut Pasteur de Côte d'Ivoire, Abidjan, Lagunes, Côte d’Ivoire; 4 Clinical Research Department, London School of Hygiene & Tropical Medicine, London, United Kingdom; 5 Noguchi Memorial Institute for Medical Research, University of Ghana, Accra, Ghana; 6 Centre Pasteur du Cameroun, Yaoundé, Cameroon; 7 National Program of African Trypanosomiasis Elimination, Abidjan, Côte d’Ivoire; 8 Mast Diagnostica GmbH, Reinfeld, Germany; 9 Institute of International Animal Health/One Health, Friedrich-Loeffler-Institut, Federal Research Institute for Animal Health, Greifswald - Insel Riems, Germany; 10 Experimental Bacteriology Unit, Institut Pasteur de Madagascar, Antananarivo, Madagascar; 11 *Laboratory for MEMS Applications, Department of Microsystems Engineering - IMTEK*, University of Freiburg, Freiburg , Germany; 12 Professorship for One Health/International Animal Health, Faculty of Veterinary Medicine, Justus Liebig University, Giessen, Germany; 13 Hospital for Tropical Diseases, London, United Kingdom; Mahidol Univ, Fac Trop Med, THAILAND

## Abstract

Yaws, caused by *Treponema pallidum* ssp. *pertenue*, remains a significant public health concern in tropical regions of West Africa and the South Pacific, primarily affecting children in remote areas with limited access to hygiene and sanitation. In this study, conducted in three endemic countries of West Africa where yaws remains a significant public health concern (Ghana, Cameroon, and Côte d’Ivoire), we aimed to assess the knowledge, attitudes, and practices related to yaws among community members, community health workers (CHWs), and traditional healers. The study revealed variations in the perception of causes of yaws among community members: the majority or participants in Ghana attributed yaws to germs (60.2%); in Cameroon the most reported form of transmission was contact with or drinking infected water sources (44.6%); and in Côte d’Ivoire both of these answers were also the most prevalent (60.3% germs and 93.% water sources). A substantial proportion of participants in Côte d’Ivoire also associated yaws with witchcraft and divine punishment (44.8%). Only a small proportion of individuals in Ghana and Côte d’Ivoire correctly identified contact with an infected person as a form of transmission (11.9% and 20.7%, respectively) and less than half in Cameroon (42.6%), although more than 98% of all participants reported avoidance behaviours towards yaws infected people due to fear of getting infected. Most participants expressed a preference for seeking care at hospitals (49.2%, 60.6%, 86.2%) or health care professionals including doctors and nurses (58.5%, 41,5% and 17.2%) if they were diagnosed with yaws, although a quarter of participants in Côte d’Ivoire also sought support from traditional healers. The CHWs interviewed were generally well-trained on yaws causes and treatment options, although they often reported low availability of treatment and diagnostic tests for yaws. Our findings underscore the need for community education, awareness campaigns, ongoing CHW training, and improved access to yaws treatment and diagnostic resources. The data also suggest that collaboration with traditional healers, who usually hold a highly esteemed position in the society, such as giving training on yaws causes and transmission or exchanging knowledge on treatment options, could be beneficial in certain regions, particularly in Côte d’Ivoire.

## Author summary

Yaws is a non-venereal ulcerative disease caused by the bacterium *Treponema pallidum* ssp. *Pertenue*. This disease is spread through skin-to-skin contact by someone with an infectious ulcer and is endemic in 13 countries, including Ghana, Cameroon and Ivory Coast. Oral azithromycin is the preferred treatment for yaws and the World Health Organization is currently deploying mass drug administration campaigns of azithromycin with the aim of global eradication of yaws. A cornerstone of this eradication campaign is reaching a population treatment coverage of over 90%, therefore education and awareness of the disease’s natural history and management is paramount. In this study, we aimed to explore knowledge, attitudes and practices regarding yaws in Ghana, Côte d’Ivoire and Cameroon to understand the general understanding of the disease and the practices around it. We found that the route of transmission of yaws is generally not well understood, with most individuals incorrectly reporting sources like dirty drinking or washing water, and supernatural causes such as curses or witchcraft. Most respondents would attend hospitals or clinics if they had yaws for correct management, although traditional healers were also a popular choice. Community health workers reported a correct management of the disease, although low stock of tests and drug availability was reported.

## Introduction

Yaws is a non-venereal treponemal infection caused by the bacterium *Treponema pallidum* ssp. *pertenue*. This chronic and recurrent disease mainly affects children living in warm, humid equatorial regions of Africa, Southeast Asia, and the Pacific [1]. Factors such as poverty, socio-economic insecurity, and poor personal hygiene are believed to contribute to the spread of yaws [2] and children under 15 years of age constitute 75–80% of the affected population, with those aged 6 to 10 being at the highest risk [1,3].

Yaws is generally transmitted by direct skin-to-skin contact during childhood with a reported incubation period between 9 to 90 days (mean 21 days) [4,5]. Yaws can appear in clusters such as in schools or hamlets, and its incidence is usually higher during rainy seasons [3]. If left untreated, yaws can lead to severe deformities, mutilations, and disabilities, highlighting the urgency of effective interventions and management [6].

The World Health Organization (WHO) leads a global eradication campaign based on the treatment of all individuals from endemic communities with azithromycin, regardless of their disease status [7]. In 2021, 124,687 suspected yaws cases were reported to WHO, most of them from Papua New Guinea (92,856 cases) and the West African Region (9,384 cases) including Ghana (3,367 cases), Congo (1,871), Central African Republic (1,218), Togo (1,028), the Democratic Republic of the Congo (669), Cameroon (647), Timor-Leste (204), Benin (198), Côte d’Ivoire (106), and Angola (76) [8]To guide eradication efforts, it is important to investigate and address local knowledge and management of yaws in endemic communities.

This study aimed to evaluate the knowledge, attitudes, and practices towards yaws of community members, community health workers (CHWs) and traditional healers in three sub-Saharan African countries: Ghana, Cameroon, and Côte d’Ivoire. These countries were selected based on their known endemicity of yaws, as documented by WHO and health authorities ([8]. The study was nested within a larger multi-country evaluation of a point-of-care diagnostic test for yaws implemented in Ghana, Cameroon and Côte d’Ivoire [9].

## Methods

### Ethics statement

We obtained ethical approval from the London School of Hygiene & Tropical Medicine (LSHTM) ethics committee (Reference: 21633, 19/08/2021) as well as local and national committees in each country. We were granted approval to conduct the study from Cameroon’s National Ethics committee for Human Research (N°2020/12/1327/CE/CNERSH/SP) and administrative authorization of the ministry of public health (N°D30-308/L/MINSANTE/SG/DROS), the National Research Ethics Committee in Côte d’Ivoire (16/09/2020), and in Ghana by the Noguchi Memorial Institute for Medical Research NMIMR institutional review board (06/11/2020) and Ghana health service (29/04/2021) National Research Ethics Committee [9]. Written consent was obtained for all individuals interviewed and no minors were included in the study.

### Questionnaires and data collection

Social scientists from each of the three participating countries developed a common questionnaire addressing key themes for each of the populations being interviewed. Themes focused on: perception and help-seeking practices for community members, yaws transmission and management for CHWs, and transmission and treatment for traditional healers.

Interviews were conducted by qualified social scientists from each of the three countries and the questionnaires were administered in official languages (French and English), although the interviews were sometimes conducted and translated to local or co-official languages and dialects. Questionnaires were administered using a standard digital form using an ODK data collection app on smartphones [10].

### Study setting and participant recruitment

Interviews were conducted in the following districts: for Ghana, Asuboi- Ayensuano district (Eastern region), Gboloo-Kofi—Akuapem North municipal (Eastern Region) and in Bawjiase—Awutu Senya East municipal (Central region); in Cameroon, Sangmelima, Lolodorf, and Djoum (Southern region), Bankim in the Adamawa region, and Messamena, Abong-Mbang, Lomié, Yokadouma, Mbang, Doumé, Batouri and Bétaré-Oya (East region); and in Côte d’Ivoire Tiassalé district (South region), Divo (West region) and Yamoussoukro district (Central region).

The recruitment strategies varied slightly across the three countries. In Ghana, community members were randomly selected from yaws endemic districts, and CHWs were directly identified by local health district offices. The search for traditional healers revealed that this practice was perceived as obsolete by community members in selected areas, attributed to the availability of health centers. Although one traditional healer was identified, the team could not find him.

In Cameroon, community members were recruited based on their yaws history and identified through the village chief or referrals from other participants during interviews. CHWs were directly identified by health authorities and referred to the research team, while traditional healers were identified by community members during interviews.

Finally, Cote d’Ivoire employed the snowball method to recruit community members. Participants were identified during door-to-door visits and subsequently referred to other community members with yaws experience. CHWs were identified through local health authorities, and traditional healers were identified and referred to the research team by study participants during interviews.

## Results

A total of 371 respondents participated in this study, including 270 community members, 86 CHWs and 15 traditional healers.

### Community members

Of the 270 community members, 118 were in Ghana, 94 in Cameroon and 58 in Côte d’Ivoire **(Table 1).**

**Table 1:** Demographic characteristics of community members by country.

		Ghana N = 118	Cameroon N = 94	Côte d’Ivoire N = 58
**Gender N (%)**	male	47 (39.8)	57 (60.6)	41 (70.7)
	female	71 (60.2)	37 (39.4)	17 (29.3)
Age median [IQR]*		33.00 [25.00, 47.75]	66.00 [46.00, 77.00]	47.50 [37.25, 54.75]

*[IQR]: interquartile range

#### Community members’ knowledge: infectious routes

Of the 118 community members surveyed in Ghana, 45.8% reported that they knew someone who had yaws or had a history of yaws themselves, compared to 77.7% of respondents in Cameroon and 60.3% in Côte d’Ivoire. The most common reported local names for yaws were Jator or Gyator in Ghana (34.7%), Mebata in Cameroon (55.3%), and Dowe/Dohe/Lohe in Côte d’Ivoire (41.4.%) (Table 2).

**Table 2:** Community members’ knowledge of yaws infection routes.

	**Ghana** **N = 118 (%)**	**Cameroon** **N = 94 (%)**	**Côte d’Ivoire** **N = 58 (%)**
**Have you or anyone you know had yaws? (yes)**	54 (45.8)	73 (77.7)	35 (60.3)
What are the local names used for yaws? *	Jator/Gyator: 41 (34.7) Dworbu: 3 (2.5) Agyakpa: 2 (2.5) Kissi kuro: 1 (0.8)	Mebata: 52 (55.3) Batakomba: 6 (6.4) Bisende mebata: 5 (5.3) Dwang, Daba, Mouta: 4 (4.3)	Dowe/Dohe/Lohe: 24 (41.4) Bogoro: 4 (6.9) Others: 7 (12.1) NA: 23 (39.7)
What are the causes/sources of the disease?*	**Ghana** **N = 118**	**Cameroon** **N = 94**	**Côte d’Ivoire** **N = 58**
*Germs*	71 (60.2)	8 (8.5)	35 (60.3)
*Contact with someone with yaws*	14 (11.9)	40 (42.6)	12 (20.7)
*Contact with dirty water*	16 (13.6)	24 (25.5)	33 (56.9)
*Drinking dirty water*	12 (10.2)	18 (19.1)	21 (36.2)
Insect bite	0 (0.0)	2 (2.1)	6 (10.3)
*Curse*	0 (0.0)	2 (2.1)	6 (10.3)
*Punishment from God*	0 (0.0)	0 (0.0)	4 (6.9)
*Witchcraft*	0 (0.0)	2 (2.1)	16 (27.6)
*Don`t know*	37 (31.4)	19 (20.2)	5 (8.6)
**Can yaws be cured?** (yes)	116 (98.3)	93 (98.9)	57 (98.3)

* Multiple choice answers.

When asked about the perceived cause of yaws 60.2% participants from Ghana and 60.3% in Côte d’Ivoire responded *germs*, while only 8.5% of interviewees in Cameroon believed germs can carry the disease. Almost all community members respondents in Côte d’Ivoire (93.1%) and a considerable proportion of respondents in Cameroon (44.6%) incorrectly believed transmission to be broadly related to contact with dirty water or drinking dirty water, while only 23.8% of respondents in Ghana reported dirty water as a source of infection. Almost half (44.8%) of the respondents in Côte d’Ivoire believed that supernatural causes, mainly witchcraft but also curses or divine punishment, could cause the disease; in contrast none of the respondents in Ghana and only 4.2% of respondents in Cameroon shared this belief.

Insect bites were reported as a transmission route by 10.3% in Côte d’Ivoire, 2.1% in Cameroon and 0% of respondents in Ghana. Finally, 11.9% of participants from Ghana, 42.6% in Cameroon and 20.7% in Côte d’Ivoire correctly believed that contact with someone with yaws could be a cause of infection. In all three countries, over 98% of respondents reported that they believed yaws can be cured with treatment (**Table 2).**

#### Community members´ attitudes: acceptability of clinical interventions

Participants were asked if they would accept a blood sample or an ulcer swab being taken should they have yaws: for Ghana, 77.1% and 78.8% reported agreement for fingerpick blood samples or lesion swabs respectively, 93.6% and 95.7% in Cameroon, and 98.3% and 100% in Côte d’Ivoire. Those who answered they would not agree were asked to explain the reasons, with the main barrier determined being fear of pain, or simply not wanting their samples being taken (**Table 3).**

**Table 3:** Community members’ attitudes and practices towards yaws.

Community members	Ghana N = 118 (%)	Cameroon N = 94 (%)	Côte d’Ivoire N = 58 (%)	
**Where would you seek care? (multiple choice answer)**				
*Doctor*	57 (48.3)	8 (8.5)	2 (3.4)	
*Hospital*	58 (49.2)	57 (60.6)	50 (86.2)	
*Nurse*	12 (10.2)	31 (33.0)	8 (13.8)	
*Traditional healer*	4 (3.4)	9 (9.6)	14 (24.1)	
*Family*	6 (5.1)	4 (4.3)	1 (1.7)	
*Pharmacy*	1 (0.8)	0 (0.0)	3 (5.2)	
*Shop*	*0 (0*.*0)*	*1 (1*.*1)*	*1 (1*.*7)*	
What are the most important factors that influence choice of where to seek care? *				
*I can get help there*	109 (92.4)	80 (85.1)	51 (87.9)	
*Cost of travel*	13 (11.0)	2 (2.1)	2 (3.4)	
*Cost of treatment*	5 (4.2)	2 (2.1)	5 (8.6)	
*Previous Experience*	3 (2.5)	12 (12.8)	12 (20.7)	
*Time*	24 (20.3)	1 (1.1)	1 (1.7)	
**Would you be happy for you/your child to have a fingerprick blood sample taken to check if you had yaws? (yes)**	91 (77.1)	88 (93.6)	57 (98.3)	
**Would you be happy for you/your child to have a swab taken from a skin ulcer/lump to check if you had yaws? (yes)**	93 (78.8)	90 (95.7)	58 (100.0)	
**How would you feel if you/your child was diagnosed with yaws?**			
Not at all worried	32 (27.1)	23 (24.5)	19 (32.8)
A little worried	12 (10.2)	21 (22.3)	6 (10.3)
Quite worried	18 (15.3)	7 (7.4)	12 (20.7)
Very worried	56 (47.5)	43 (45.7)	21 (36.2)
**Do members in the community avoid people with yaws? (yes)**	4 (3.4)	44 (46.8)	25 (43.1)	

*Multiple choice answers

#### Community members´ attitudes: concern levels and stigma towards yaws

We reported perceptions and stigma related to yaws in all three countries. Participants’ levels of concern about having a child with yaws varied across groups: those who were *less worried* attributed this to the availability of treatment, the *moderately worried* individuals generally mentioned fear of reduced daily activity and the fear of sickness, while the *considerably worried* group mentioned pain, high treatment costs, and concerns about disease progression. The *very worried* group of participants emphasized fears of disease spread, disability, and progression to leprosy (this was mostly mentioned by Côte d’Ivoire respondents) (**Table 3).**

Stigma toward yaws-affected individuals differed among countries; in Ghana only 3.4% of participants reported avoidance behaviour, whereas 43.0% in Cameroon and 46.0% in Côte d’Ivoire reported community avoidance tendencies. Despite these variations, all who reported avoidance or stigma of the community towards yaws explained that it was due to fear of infection spreading.

#### Community members´ practices: care seeking

When asked about care seeking practices, many individuals reported they would attend a hospital should they ever have yaws (49.2% Ghana, 60.6% Côte d’Ivoire, 86.2% Cameroon). Visiting a doctor or a nurse was also commonly reported (58.5%, 41,5% and 17.2%). A quarter (25.8%) of participants in Côte d’Ivoire also reported they would attend a traditional healer compared to 9.6% in Cameroon and a 3.4% in Ghana. The main responses to choosing each caregiver were knowing they could be helped there, previous experiences, and time to get there (**Table 3**).

### Community Health Workers (CHWs)

Overall, 85 CHWs were surveyed; five in Ghana, 54 in Cameroon, and 26 in Côte d’Ivoire (**Table 4).**

**Table 4:** Community Health Workers’ (CHW) knowledge on yaws infection routes.

CHW	Ghana N = 5	Cameroon N = 54	Côte d’Ivoire N = 26
**Did CHW know about yaws?**	Yes: 5	Yes: 4 (No: 50)*	Yes: 26
**How does yaws spread? (Multiple choice question)**	Contact: 5 Contact dirty water: 3	Contact: 2 Contact dirty water: 1 Insect bite: 1	Contact: 19 Dirty water: 8 Drinking dirty water: 2 Insect: 1 Other: 7

*These 50 CHWs did not continue the questionnaire.

#### Community Health Workers’ knowledge: yaws transmission

All CHWs surveyed in Ghana and Ivory coast reported that they had seen someone with yaws before, but 50 (92,6%) CHWs in Cameroon reported they had never heard of yaws before. Only those who reported knowing about yaws were asked to complete the questionnaire. When asked about the factors involved in the transmission of this disease, CHWs’ answers varied from country to country. In Ghana, the five CHWs (100%) interviewed correctly reported that yaws is transmitted by contact with someone with yaws. In Cameroon, two CHWs (50%) reported contact with someone, while one responded contact with dirty water, and another one reported insect bite. In Côte d’Ivoire, 19 (73.1%) CHWs reported contact with someone with yaws, eight (30.8%) reported contact with dirty water, two (7.7%) reported drinking dirty water and one (3.8%) CHW associated infection with insect bites (Table 4).

#### Community Health Workers’ knowledge: yaws case management, treatment and testing

When asked about treatment options, in Ghana all five interviewed CHW reported using azithromycin tablets as a treatment for yaws, while in Cameroon, all four CHWs reported using benzathine penicillin G injection. In Côte d’Ivoire, 12 CHWs used azithromycin, 7 used penicillin, and 7 reported using other treatments.

Regarding drug availability, two out of five CHWs in Ghana confirmed having the necessary drugs in stock, while three reported stockouts. In Cameroon, all five CHWs confirmed regular availability of drugs. In Côte d’Ivoire, 13 CHWs reported having the drugs in stock, nine CHWs experienced regular stockouts, and four CHWs did not provide an answer.

All CHWs in Ghana reported conducting regular contact tracing for yaws, in Cameroon three CHWs engaged in contact tracing, and in Côte d’Ivoire 17 reported this practice. In terms of responsibility, all five CHWs in Ghana reported that health workers oversaw contact tracing after a yaws patient is identified, while in Cameroon three CHWs indicated that community health volunteers (CHV) handled contact tracing, and one did not respond. In Côte d’Ivoire, eight CHW (30.8%) reported the responsibility fell on themselves, while 9 reported this is responsibility of the CHV; nine did not answer.

Regarding the type of diagnostic test used, in Ghana, two CHWs reported referring samples for PCR testing at national level while the remaining three employed RPR (rapid plasma reaging) blood tests. In Cameroon, one CHW reported PCR referral, one used RPR and two reported using RDTs. In Côte d’Ivoire, a total of 14 CHWs utilized blood tests for diagnostic confirmation RPR or TPPA (Treponema pallidum particle agglutination), four reported using RDTs (Rapid diagnostic tests) (15.4%) and eight (30.8%) reported referring for PCR analysis. All CHW reporting the use of PCR also reported sending the samples away to a central laboratory for analysis. Overall availability of diagnostic tests in stock was 25% in Cameroon (1 CHW), 40% in Ghana (2 CHW) and 38.5% in Côte d’Ivoire (10 CHWs) **(Table 5).**

**Table 5:** Community Health Workers´ yaws case management.

CHW	Ghana N = 5	Cameroon N = 4	Côte d’Ivoire N = 26
Do you use AZT* or BP** to treat yaws?	AZT: 5	BP: 4	AZT: 12 BP: 7 Other: 7
**Do you have these drugs in stock?**	Yes: 2 No: 3	Yes: 4	Yes: 13 No: 9 NA:4
**Do you do contact tracing**	Yes: 5	Yes: 3 No: 1	Yes: 17 No: 9
**Who is in charge of contact tracing**	HCW: 5	CHV: 3 NA: 1	HCW: 8 CHV: 9 NA: 9
**Do you have tests for yaws**	No: 5	No: 4	Yes: 10 No: 15 NA: 1
**Which test do you usually use?**	Swab: 2 Blood: 3	RDT: 2 Swab: 1 Blood: 1	RDT: 4 Swab: 8 Blood: 8 TPPA: 6
**Do you send the samples or tests to other lab**	Yes: 2 No: 3	Yes: 1 No: 3	Yes: 2 No: 24
**Do you usually have tests on stock**	Yes: 2 No: 3	Yes: 1 No: 3	Yes: 10 No: 15 NA: 1
**What is your personal reaction to yaws**	Quite worried: 3 Very worried: 2	Little worried: 3 Not worried: 1	Not Worried: 16 Little Worried: 4 Quite Worried: 4 Very Worried: 2

*AZT: azithromycin

**BP: Benzathine penicillin G

When we asked CHW about how concerned they would be if they got infected with yaws, their level of concern and stigma followed similar trends to those reported by community members.

"It is a bad disease because it can prevent you from continuing my studies, it can paralyze me, it can keep me away from society because it’s contagious **(29 years old, Man; Community health worker in Côte d’Ivoire)".**

"I know there’s a treatment, but like any disease, I’m a little worried about whether the treatment will work, and whether there are any side effects **(57 years, Woman; Community health worker Côte d’Ivoire)".**

### Traditional healers

In Cameroon, we interviewed eight traditional healers, with six being female from the Boulou ethnic group and identified as Catholic. Half of them believed yaws to be transmitted through contact with an infected person, while the other half were uncertain about transmission. All traditional healers used forest-found herbs for yaws treatment. In Côte d’Ivoire, all eight traditional healers surveyed were male, from the Baloue and Senoufo ethnicities, also having Catholic beliefs. While four traditional healers attributed yaws to supernatural causes, the others associated it with contact with dirty water. In Côte d’Ivoire, only one traditional healer employed modern drugs for treatment, while most relied on traditional remedies like ash, lemon, and forest-sourced herbs. No traditional healers were interviewed in Ghana.

## Discussion

The study shows variability on knowledge, attitude and practices regarding yaws amongst the three countries and the groups interviewed. Although statistical tests were not performed, we report differences in some of the reported knowledge and practices regarding this disease. To our knowledge, this is the first study reporting yaws experiences in communities from Cameroon and Côte d’Ivoire and the second in Ghana.

Despite Ghana having a higher prevalence of yaws compared with the other two countries [8], less than half (45.8%) of participants reported having had yaws or knowing someone who had it, while in comparison, Cameroon and Côte d’Ivoire, with comparatively lower yaws prevalence, had higher percentages of participants who reported having had yaws or having known people with yaws (77.7% and 60.3%, respectively). This observed difference is likely linked to a sampling bias: in Ghana, community members were randomly selected, while in the other countries they were selected based on their reported experience with yaws, although it could also be attributed to factors such as misdiagnosis by presence of other similar skin conditions that may be mistaken for yaws (Buruli ulcer, haemophilus ducreyi, leishmaniasis), leading to incorrect associations. During fieldwork activities, the teams visited numerous participants who had initially been reported to have yaws but were subsequently diagnosed with alternative ulcerative diseases.

Furthermore, the average age of community members interviewed in Ghana was markedly higher than in Cameroon and Côte d’Ivoire likely leading to different educational and disease awareness levels. The observed disparity in age can be also attributed to the different sampling methodologies employed by each country. In Ghana, random selection potentially introduced bias towards the inclusion of older individuals who were present in the village during working hours, while in Cameroon and Côte d’Ivoire, individuals were purposefully targeted based on their experience with yaws, leading to a higher likelihood of engaging with younger adults, particularly parents of children affected by yaws.

The majority of community members’ respondents in Ghana (60.2%) and Côte d’Ivoire (60.3%) associated yaws with "germs," suggesting some awareness of its infectious nature. This is consistent with a previous report from Ghana by Marks et al, where 77% of participants also reported germs as a source of yaws [11]. However, there were misconceptions about transmission routes; these mainly included contact with or drinking dirty water, and in the case of Côte d’Ivoire, a strong inclination for supernatural causes like witchcraft, curses or punishment from God (44.8%). No reports of supernatural causes were identified in Ghana or Cameroon. Almost half (42.6%) of the Cameroonian respondents correctly identified contact with another person as a potential way to transmit yaws, this was true for 20.7% participants in Côte d’Ivoire and for only 11.9% of participants from Ghana, which differs largely from the 45.8% reported by Marks in Ghana [11]. The different results in Ghana could again be due to different sampling techniques in both studies or on the questionnaire implementation or layout, but in general the results across all three countries reflect a general lack of awareness about the contagious nature of the disease through direct skin-to-skin contact which contrast with the high reported stigma and avoidance behaviours towards infected people which are reportedly due to fear of getting infected.

Most respondents across all three countries expressed their intention to seek care at hospitals or clinics if they or their children were diagnosed with yaws. Furthermore, almost all respondents reported a very high acceptability for diagnostic procedures. This inclination towards formal healthcare facilities and diagnosis is promising for yaws eradication efforts and reflects confidence in the healthcare system. However, a noteworthy proportion of participants in Côte d’Ivoire expressed a preference for traditional healers who reportedly do not use standard yaws treatment such as penicillin or azithromycin. These results from Côte d’Ivoire are consistent with the supernatural beliefs regarding transmission routes discussed above.

Overall, supernatural beliefs, as reported in Côte d’Ivoire are commonly reported in these countries for other skin diseases such as leprosy or Buruli in Cameroon and Ghana suggesting that stigmatization and discrimination towards skin NTDs must be addressed in these regions through regular community and school based educational campaigns and by delivering information at health centres. Misconceptions on infectious routes are also common for other skin NTDs, which furthers reinforces the need for educational campaigns [12–14].

CHWs play a crucial role in yaws eradication efforts by providing knowledge and healthcare services to their communities. While the majority of CHWs were educated on yaws transmission and treatment practices, challenges such as treatment and diagnostic test availability were often reported as well as irregular contact tracing reporting. Contact tracing and follow up of confirmed cases are key components of the eradication strategy [7], and health authorities should aim to a strict implementation and high coverage of these practices to effectively eradiation the disease. Some of the CHWs in Côte d’Ivoire and Cameroon reported using RDTs for yaws diagnosis, although it is unlikely these are DPP rapid tests (Chembio’s Dual Pathway Platform syphilis test) as they are still rarely available in remote areas. The only other rapid test option is the Syphilis RTD, a treponemal test that cannot confirm an active yaws infection [15]. These reports from the CHWs could reflect a weak implementation of the yaws eradication guidelines including identification, treatment and contact tracing in these areas. Ensuring continuous training on yaws diagnosis and management for CHWs is vital for effective yaws management as well as ensuring stock and availability of diagnostic tests and yaws treatment (azithromycin and benzathine penicillin G) on site. Azithromycin for cases and contacts should be available for free in these three countries as treatment is accessible to health authorities through WHO and donor organizations. Availability of reliable diagnostic tests, notably rapid non-treponemal tests (DPP) or PCR testing facilities available remotely is essential to assess the actual impact of yaws in these communities given that clinical diagnosis is very unreliable; recent studies have shown that a significant proportion of yaws-like ulcers identified in yaws endemic settings are in fact caused by *Haemophilus ducreyi* and a range of other ulcerative pathogens, most of which, though not all, respond to azithromycin treatment [16,17]. Confirming these diverse causative agents requires PCR tests, which are rarely available on site.

Traditional healers are continuously identified as occupying distinctive roles of trust for NTD management within their communities [11,18]. In this study we report that the few interviewed traditional practitioners occasionally attribute yaws to supernatural causes, and they always resort to traditional remedies for therapeutic interventions that are unlikely to be effective against yaws. The results Cote d’Ivôire in this study and of other studies on NTDs in West Africa, suggest that many communities rely strongly on traditional healers as their caregivers [13]; enhancing the education and involvement of traditional practitioners in collaboration with the formal healthcare sector could benefit yaws eradication efforts as some populations,

Finally, yaws is often found to be co-endemic with several other skin NTDs and other skin diseases, such as scabies, leprosy or Buruli ulcer, that are caused under similar conditions and require similar management and care approaches [19]. Therefore, integrated management by targeting several skin NTDs should always be planned during awareness or treatment campaigns in order to save costs and further benefit the population by offering a wide range of skin disease clinical diagnosis through these visits [20].

We acknowledge certain limitations that call for consideration in the interpretation of findings. First, the sample size of CHWs and traditional healers involved in the study is small, and we suggest caution in drawing broad conclusions. An additional limitation is the uneven distribution of sample sizes and demographic representation of community members across countries potentially due to the previously discussed recruitment bias or different recruitment techniques between the three countries, which impedes statistical comparisons between countries.

## Conclusion

This study provides insights into the knowledge, attitudes, and practices regarding yaws in endemic areas of Ghana, Cameroon, and Côte d’Ivoire. Understanding these factors is essential for tailoring yaws eradication strategies to specific country-based and community needs. Culturally sensitive health education campaigns that promote personal hygiene as the main way to prevent yaws and other skin NTDs together with ensuring availability of drugs and diagnostic tests are essential components of comprehensive yaws eradication efforts in these regions. Furthermore, ensuring the consistent training of CHWs and collaboration with traditional healers can strengthen community-based interventions and facilitate early diagnosis and treatment, ultimately contributing to the goal of yaws eradication. Yaws eradication efforts must be addressed in combination with the management of other skin NTDs in an intergraded approach.

## Supporting information

S1 Data Full study dataset. (XLS)
